# Associations of socioeconomic status and obesity with hypertension in tibetan adults in a Chinese plateau area

**DOI:** 10.1186/s12889-023-15864-9

**Published:** 2023-09-21

**Authors:** Yajie Li, Jianbo Li, Qucuo Nima

**Affiliations:** 1 Tibet Center for Disease Control and Prevention, 21 North linkuo Road, Lhasa, Tibet, China; 2 Wuzhong District Center for Disease Control and Prevention, Suzhou City, China

**Keywords:** Socioeconomic status, Obesity, Hypertension, Additive interaction

## Abstract

**Background:**

Previous studies have identified that socioeconomic status (SES) and obesity are associated with hypertension. However, their interaction on hypertension risk has not yet been assessed.

**Methods:**

The study used data from 6,069 Tibetan residents in Chengguan District in Lhasa, the Chinese Tibetan autonomous region’s capital, based on a cohort study conducted from May 2018 to September 2019 in five provinces in southwest China. We used logistic regression models to assess the complex relations of SES and obesity with hypertension.

**Results:**

Compared with individuals of high SES, low and moderate SES were positively associated with high risk of hypertension. SES and obesity have significant additive interaction on hypertension (general obesity by BMI: RERI = 1.33, P < 0.001; abdominal obesity by WC: RERI = 0.76, P < 0.001; abdominal obesity by WHtR: RERI = 0.96, P < 0.001). In people from the low and moderate SES segments, obesity was linked to an increased risk of hypertension, but the correlations were stronger in people from the moderate SES category. Compared with people of high SES and non obese, those with moderate SES and obesity had a higher risk of hypertension, and ORs were 4.38 (2.80, 6.84) for general obesity by BMI, 3.38 (2.05, 5.57) for abdominal obesity by WC, and 3.18 (1.57, 6.42) for abdominal obesity by WHtR.

**Conclusion:**

There is an independent and additive interaction effect of obesity and SES on the risk of hypertension. People with obesity, especially those of moderate and low SES, should reduce weight and waist circumference, and pay more attention to blood pressure. Moreover, the government, health administration departments, and society should prioritize improving the socioeconomic status of the Tibetan population and addressing risk factors like obesity.

**Supplementary Information:**

The online version contains supplementary material available at 10.1186/s12889-023-15864-9.

## Introduction

The development of society and the economy has led to an increased consumption of high-fat, high-sugar, and high-sodium foods among ethnic minorities in plateau areas. Tibet, also known as the “Third Pole,“ is situated in the southwest of the Qinghai-Tibet Plateau. With an average altitude exceeding 4,000 m, the region is characterized by a harsh climate, strong radiation, low pressure hypoxia, barren land, and a poor economic foundation [1]. Of the permanent population, 86.01% (3,137,901 individuals) adopt a high-fat and high-sodium diet [2]. Furthermore, according to China’s seventh population census in 2020, the percentage of individuals with a high school education or higher in Tibet is only 18.07%, significantly lower than the national average of 30.55%^1^.This, combined with a lack of healthy lifestyle choices and health interventions, harsh natural environments with low oxygen and high altitude, and complex ethnic cultural traditions and customs, has created severe and complex health problems and behavioral risk factors [3, 4, 5]. A Chinese study found that overall health among people in the Tibet Autonomous Region is lower than that of individuals in other Chinese provinces [6]. Previous research has demonstrated that hypertension incidence in Tibet is higher than in other parts of China [7]. Furthermore, a systematic review indicated that the prevalence of hypertension in Tibetan residents increases by 2% for every 100 m increase in altitude [8]. In 2019, cardiovascular diseases (CVDs) were responsible for 46.74% and 44.26% of mortality in rural and urban areas of China, respectively [9]. Hypertension is a major risk factor for CVDs and a serious threat to human health [10]. As socioeconomic development, population aging, and urbanization accelerate, the prevalence of hypertension is increasing and has become a significant public health issue [11].

Socioeconomic status (SES) is a measure of an individual’s position in the class system, including factors such as education, income, occupation, wealth, and place of residence. SES widens health gradients between groups by providing health advantages [12, 13]. Previous studies have reported the presence of SES-health gradients and a meta-analysis found that individuals with the lowest SES in terms of income, occupation, and education had a higher prevalence of hypertension than those with the highest SES [14]. In an aging society with social stratification, individual health inequalities may tend to expand with age, known as cumulative disadvantage of health disparities. This kind of health disparity is evident in Tibet, which is sparsely populated and unevenly developed.

SES and obesity are well-known predictors of hypertension, and there is substantial evidence linking SES to traditional risk factors for hypertension such as health knowledge, behaviors, lifestyle, working conditions, medical care, income, and socio-psychological resources [15–17]. Studies on obesity have demonstrated that body fat distribution, particularly the accumulation of abdominal fat, is strongly associated with CVDs and is valuable in predicting the risk of CVDs [18]. While the relationship between SES, obesity, and hypertension is not new, it has received less attention in high-altitude regions with ethnic minorities. Furthermore, studies on hypertension and obesity among Tibetan residents in Tibet are relatively scarce.

There is a global debate on the predictive role of various obesity markers for hypertension development. Relying solely on a single obesity marker to predict hypertension may weaken the evidence’s strength. Moreover, current studies face several issues. First, although many studies have examined the relationship and pathways between obesity, hypertension, and SES, few have investigated how SES and obesity interact to cause hypertension. Second, earlier studies tended to use a single measure to reflect individuals’ overall SES level [19], indicating the need for a comprehensive SES index that can adequately reflect its effects on obesity and hypertension. Additionally, the lack of targeted studies on characteristic populations calls for further exploration of the relationship between SES, obesity, and hypertension in Tibetans.

This study aims to verify the association between SES, obesity, and hypertension in Tibetan residents to address the current research gap. The findings of this study can inform future research and aid in developing public health policies for similar high-altitude indigenous populations in China or other countries.

## Methods

### Study population

This study was conducted from May 2018 to September 2019 in five provinces of south-western regions of China. Data was collected primarily through electronic questionnaires and health examinations. Details of the study design, data collection, and methods were reported previously [20]. The ethics committee of Sichuan University (K2016038) approved this study. We obtained informed consent from all participants, and we conducted all methods in compliance with the relevant guidelines and regulations. The data used in this study was from the Chengguan District in Lhasa, the Chinese Tibetan autonomous region’s capital. For the current analyses, individuals missing data on any outcome, exposure, or adjusted variables were also disqualified, as were participants under the age of 18. In the end, 6069 people in all were used for the analyses.

### Exposure

Based on previous studies, SES was measured using self-reported family income level, employment, education level, and health insurance gathered through questionnaires [21]. Each element was separated into three levels, with each level taking into account the sample size and practical interpretation. The family income level was categorized into < 20000yuan, 20000-59999yuan, and ≥ 60000yuan. Education was categorized into illiteracy, primary school, junior high school or higher. Occupation was categorized into upper (including administrative and management personnel, professional technical personnel, retirees), lower (including agricultural, forestry, animal husbandry and fishery workers; sales and service staff) and unemployment. Health insurance was categorized into medical insurance for urban population, new rural cooperative medical system, and no health insurance.

An overall SES variable based on family income level, employment, education level, and health insurance was developed using the latent class analysis. Using item-response probabilities, three latent classes were identified, representing high, moderate, and low SES [22]. Table S1 describes the indicators in models with different numbers of latent classes.

Participants’ height, waist circumference (WC), and weight were measured anthropometrically to determine obesity according to a standard protocol from the Working Group on Obesity [23]. Obesity was assessed by BMI, WC, and WHtR indicators. The cut-off values for the BMI, WC, and WHtR for obesity were 28.0 kg/m^2^ for both sexes, 90 cm for men and 85 cm for women, and 50.0% for both men and women, respectively [24–26].

### Assessment of outcome and other covariates

Participants’ blood pressure (BP) was measured using electronic sphygmomanometers by trained medical personnel, according to the American Heart Association’s standardized protocol [27]. Diastolic BP (DBP) and systolic BP (SBP) were calculated based on the average of the three measurements. Hypertension was defined as having an average measured SBP≥140 mmHg or DPB≥90 mmHg or any use of antihypertensive medication (self-reported hypertension)[28].

The smoking status is a three-category variable (never, current, and former). Never smoking refers to individuals who have smoked fewer than 100 cigarettes in their lifetime. Current smoking was defined as having smoked more than 100 cigarettes in their lifetime, and former smoking was defined as having quit smoking for more than six months [29]. The Alcohol drinking status is a three-category variable (never, occasionally, and often). “Never drinking alcohol” means that the individual has never consumed any alcoholic beverages throughout their lifetime. Often drinking was defined as drinking alcohol more than two days per week on average over the past year, while occasional drinking was defined as drinking alcohol less than three days a week on average during the past year. The DASH score concentrated on seven different dietary categories, including fresh fruits and vegetables, legumes, whole grains, red and processed meat, dairy, and salt with a sodium content, and each category was given a score from 1 to 5 based on the quintile of the average food consumption [30, 31]. Physical activity comprehensively considered the occupation, transportation, household, and leisure time of participants [32]. Sleep disorders were assessed by early awakening, difficulty in falling asleep, drug-assisted sleep and poor sleep, and any positive one was defined as sleep disorder [33].

### Statistical analysis

The basic characteristics of hypertensive and normotensive individuals were described using descriptive statistics. Continuous and categorical characteristic variables between normal and hypertensive subjects were reported as mean ± SD (The statistical test results show that the data presents a normal distribution) and numbers (percentages) and compared using the Student’s t-test and chi-squared test.

Multivariable logistic regression model was used to evaluate the association of SES and obesity on hypertension. Based on the previous literature [34], fully adjusted models including the following covariates: age, sex (male and female), smoking status (never, current, and former), alcohol status (never, occasionally, and often), physical activity, DASH score, sleep disorder (yes and no), and hypertension family history (yes, no, and not sure).

We conducted a stratified analysis by latent class of SES to explore associations of obesity with hypertension among adults in different socioeconomic subgroups. In this analysis, participants with normal weight served as the reference group, and we examined whether obesity was associated with an increased risk of hypertension in various SES categories.

We also included a product term for SES (low and high) and obesity (yes and no) to the model in order to quantify the additive and multiplicative interactions. The odds ratio (OR) with its 95% confidence interval (CI) of the product term was the measure of interaction on the multiplicative scale. We used the relative excess risk due to interaction (RERI) and its corresponding 95% CI as the measure of interaction on the additive scale.

We further divided people into six groups based on SES (low, moderate, and high) and obesity (yes or no) and computed ORs of hypertension in each group compared with those with high SES and normal weight in order to investigate the combined correlations.

We performed several sensitivity analyses. First, we additionally adjusted for diabetes to minimize the influence of multimorbidity. Second, we excluded those had any physician-diagnosed hypertension to test the accuracy of the effect estimates. Third, we used the higher cutoff point of BMI ≥30 (the recommendations of the World Health Organization) to test the stability of the results. R 4.0.2 (R Foundation for Statistical Computing) was used for the analyses, and statistical significance was declared if P values < 0.05.

## Results

### Population characteristics

Table 1 presents the general characteristics of the study population, including their average age of 46.97 years, with 60.70% being female. The population consisted of 6,069 participants, of which 381 (6.28%) had high SES, 1,556 (25.64%) had medium SES, and 4,132 (68.08%) had low SES. Hypertensive participants tended to be older, male, former smokers, frequent drinkers, less physically active, have a lower DASH score, lower SES level, a family history of hypertension, and experience sleep disorders. Moreover, the hypertensive participants had higher BMI, WC, and WHtR obesity indicators compared to the normotensive participants.

**Table 1:** Basic characteristics of study participants

Characteristics*	Total (n = 6069)	Normotension (n = 4334)	Hypertension (n = 1735)	P-value
Age, years (SD)	46.97(12.86)	43.92(12.10)	54.57(11.49)	< 0.001
DASH score (SD)	20.55(3.43)	20.66(3.40)	20.27(3.49)	< 0.001
Physical activity, METs/d (SD)	20.96(16.64)	21.50(16.84)	19.61(16.05)	< 0.001
WHtR, % (SD)	57.02(7.53)	55.87(7.34)	59.88(7.25)	< 0.001
WC, cm (SD)	91.63(11.82)	89.94(11.60)	95.85(11.30)	< 0.001
BMI, kg/m^2^ (SD)	25.35(3.51)	24.95(3.39)	26.34(3.60)	< 0.001
Sex, n (%)				< 0.001
Male	2385(39.30)	1605(37.03)	780(44.96)	
Female	3684(60.70)	2729(62.97)	955(55.04)	
Socioeconomic status (%)				< 0.001
High SES	381(6.28)	275(6.35)	106(6.11)	
Medium SES	1556(25.64)	1239(28.59)	317(18.27)	
Low SES	4132(68.08)	2820(65.07)	1312(75.62)	
Smoking status, n (%)				< 0.001
Never	4620(76.12)	3333(76.90)	1287(74.18)	
Former	297(4.89)	169(3.90)	128(7.38)	
Current	1152(18.98)	832(19.20)	320(18.44)	
Alcohol drinking status, n (%)				< 0.001
Never	4146(68.31)	2980(68.76)	1166(67.20)	
Occasionally	1521(25.06)	1145(26.42)	376(21.67)	
Often	402(6.62)	209(4.82)	193(11.12)	
Hypertension family history, n (%)				0.002
Yes	1507(24.83)	1042(24.04)	465(26.80)	
Not sure	2183(35.97)	1533(35.37)	650(37.46)	
No	2379(39.20)	1759(40.59)	620(35.73)	
Sleep disorder, n (%)				< 0.001
Yes	2033(33.50)	1365(31.49)	668(38.50)	
No	4036(66.50)	2969(68.50)	1067(61.50)	

BMI: body mass index; DASH: dietary approaches to stop hypertension; METs: metabolic equivalent tasks; WC: waist circumference; WHtR: waist-to-height ratio; SES: socioeconomic status

*Data are the mean (SD) for continuous variables and number (percentage) for categorical variables

### Association of SES and obesity with hypertension

Table 2 displays the ORs and 95% CIs for the association of obesity and SES with hypertension according to adjusted models. The results indicate a significant association between SES and hypertension, with an OR of 1.97 (1.51, 2.57) when participants of low SES were compared to those of high SES, after controlling for age, sex, smoking status, alcohol drinking status, physical activity, sleep disorder, DASH score, hypertension family history, and general obesity (by BMI). Positive associations were also observed between obesity indicators and hypertension, with ORs of 1.95 (1.67, 2.27), 1.92 (1.66, 2.21), and 2.12 (1.74, 2.58) for BMI, WC, and WHtR indicators, respectively. Sensitivity analyses produced identical results (see Table S2 and Table S3).

**Table 2:** Associations of socioeconomic status and obesity with hypertension

Variables	Model 1	Model 2	Model 3
Socioeconomic status			
High SES	1(Reference)	1(Reference)	1(Reference)
Medium SES	2.16(1.60, 2.92)	1.90(1.41, 2.56)	1.93(1.43, 2.60)
Low SES	1.97(1.51, 2.57)	1.76(1.35, 2.29)	1.76(1.35, 2.30)
Obesity			
No	1(Reference)	1(Reference)	1(Reference)
Yes	1.95(1.67, 2.27)	1.92(1.66, 2.21)	2.12(1.74, 2.58)

SES: socioeconomic status. All models adjusted for age, sex, smoking status, alcohol drinking status, physical activity, sleep disorder, DASH score and hypertension family history. Model 1 was for general obesity by BMI; Model 2 was for abdominal obesity by waist circumference; Model 3 was for abdominal obesity by waist-to-height ratio

### Interaction and joint analysis of SES and obesity with hypertension

**Fig. 1:**
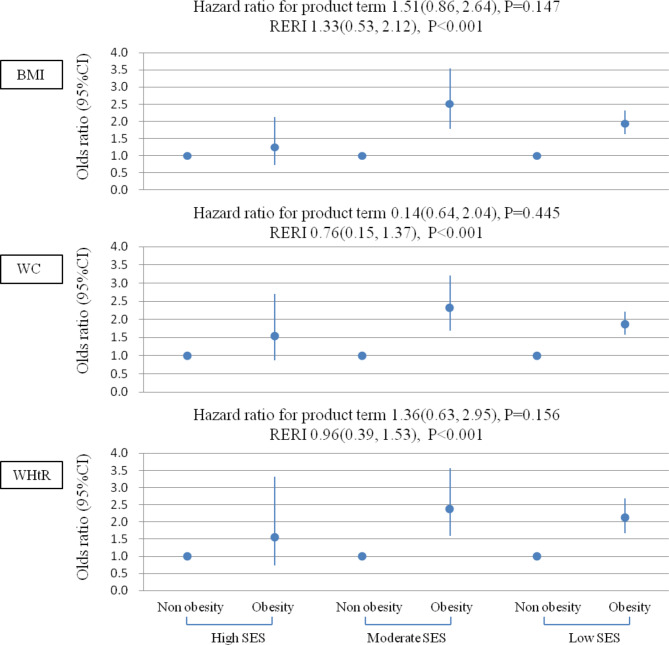
Associations of obesity with hypertension by socioeconomic status BMI: body mass index; WC: waist circumference; WHtR: waist-to-height ratio; SES: socioeconomic status

A significant additive interaction between SES and obesity (general obesity by BMI, abdominal obesity by WC and WHtR) on hypertension was found, but there was no significant multiplicative interaction (P > 0.05). For instance, when comparing participants of low SES to those of high SES, the RERI was 1.33 (0.53, 2.12), indicating a relative excess risk of 1.33 due to the additive interaction (see Fig. 1).

All models adjusted for age, sex, smoking status, alcohol drinking status, physical activity, sleep disorder, DASH score and hypertension family history.

Multiplicative interaction was evaluated using hazard ratios for the product term between obesity (yes *v* no) and SES (low *v* high), and the multiplicative interaction was statistically significant when its confidence interval did not include 1. Additive interaction was evaluated using relative excess risk due to interaction (RERI) between obesity (yes *v* no) and SES (low *v* high), and the additive interaction was statistically significant when its confidence interval did not include 0.

The study found that individuals in the low and moderate SES groups had an increased risk of hypertension linked to obesity, with the correlations being stronger in the moderate SES group. For instance, the ORs for hypertension among individuals with general obesity by BMI compared to normal weight were 1.24 (0.73, 2.13) for those in the high SES group, 2.50 (1.77, 3.54) for those in the moderate SES group, and 1.94 (1.62, 2.32) for those in the low SES group (Fig. 1).

**Fig. 2:**
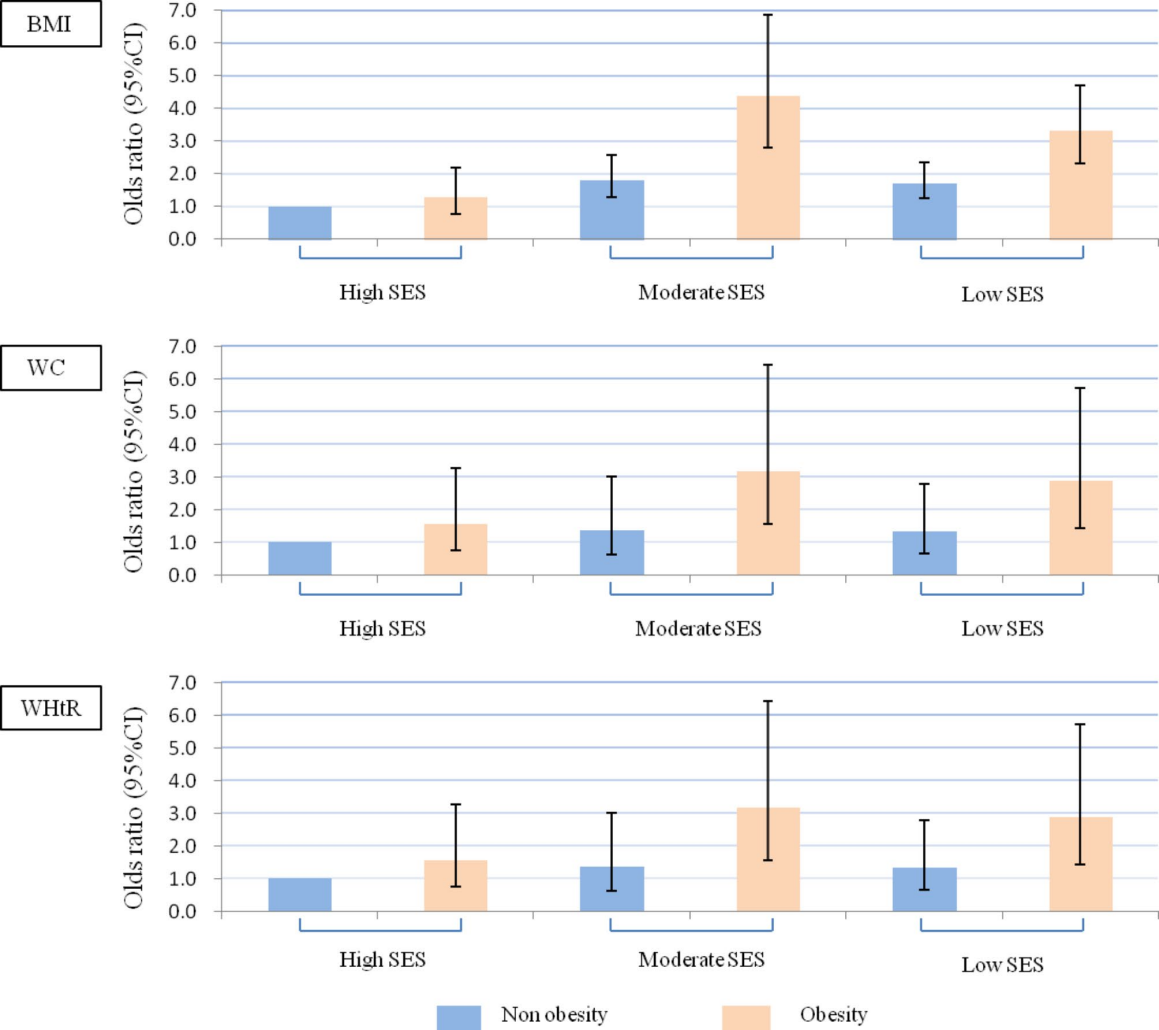
Joint associations of socioeconomic status and obesity with hypertension BMI: body mass index; WC: waist circumference; WHtR: waist-to-height ratio; SES: socioeconomic status.

Figure 2 illustrated the combined association of SES and obesity with hypertension. The results showed that adults with moderate SES and obesity had a higher risk of developing hypertension than those with high SES and no obesity, with ORs of 4.38 (2.80, 6.84) for general obesity by BMI, 3.38 (2.05, 5.57) for abdominal obesity by WC, and 3.18 (1.57, 6.42) for abdominal obesity by WHtR (Fig. 2).

All models adjusted for age, sex, smoking status, alcohol drinking status, physical activity, sleep disorder, DASH score and hypertension family history.

## Discussion

An independent and additional interaction effect of obesity and SES on hypertension was observed. Positive associations were found between lower SES and hypertension, with stronger correlations of obesity with hypertension among those of moderate SES. The highest risks of hypertension were observed in people with moderate SES and obesity. This is the first study to examine the interaction associations of three types of obesity and overall SES with hypertension.

In previous studies, a single variable such as income, occupation, or education level was typically used to represent SES at the individual level. Studies investigating the relationship between SES and hypertension have reported mixed results, with some finding a positive association [35, 36] and others reporting a negative association [37, 38]. A population-based cohort study conducted in rural South-Central Uganda found that hypertension was more prevalent among those with higher SES [37], while a study of 76 low- and middle-income countries (LMICs) found that hypertension was more common among adults in the lowest socioeconomic classes as LMICs developed economically [36]. The discrepancy in findings across previous studies may be due to a variety of factors, including socioeconomic inequality and social patterns of behavior that contribute to risk factors [39]. For example, evidence suggests that individuals of low SES in LMICs are more likely to use tobacco and alcohol and to consume a less healthy diet, while individuals of high SES tend to be more physically inactive [40]. Additionally, most studies have investigated only a single socioeconomic factor, but different socioeconomic factors represent various aspects of SES or social class and should not be simply interchanged with one another [19]. Our study found a positive association between low and moderate SES and a higher risk of hypertension compared to participants of high SES. The relatively low levels of economic and educational development in Tibet are important factors that impact the prevention and treatment of hypertension.

Our study found a positive correlation between hypertension and all three types of obesity indicators, which is consistent with previous research [41–43]. The prevalence of obesity and associated disorders is increasing worldwide, with adipose storage accounting for 60% of hypertension cases [44]. The relationship between obesity and hypertension is complex and multifactorial, involving genetics, environment, sympathetic nervous system activity, renal function, and insulin resistance [45]. In Tibet, the cold climate has led to a diet high in fat, sodium, and protein, and low in sugar and cellulose, with staples such as yak beef, milk residue, ghee, and sweet tea [46]. This diet may contribute to the dual threat of obesity and hypertension among Tibetans.

Our study confirmed that all three types of obesity indicators were associated with higher risks of hypertension among participants with moderate and low SES. Additionally, we found significant additive interactions, with those with moderate SES showing a stronger link between obesity and hypertension. These findings emphasize the importance of weight loss and waist circumference reduction, particularly for those with moderate SES. Few studies have examined the interaction between SES and obesity on the risk of hypertension. One study conducted in Peru showed that education level was an effect modifier of the association between BMI and both SBP and DBP [47]. The socioeconomic distribution of the most significant non-communicable disease risk factors may be related to the biological mechanism of the interaction between SES, obesity, and hypertension [40], and this interaction may also be affected by the plateau environment and Tibetan dietary habits. Compared to people with low SES, those with moderate SES may be more likely to consume a high-fat diet, while those with low SES tend to be more physically active [48]. These are important influencing factors for the prevalence of hypertension. These findings underscore the need for weight loss and waist circumference reduction, particularly for those with moderate SES. To fully understand the intricate interactions between SES, obesity, and hypertension, further research is still required.

This is the first study reporting a significant additive interaction between SES and obesity on the risk of hypertension. An overall SES variable was created to assess the complex relationships between SES, obesity, and hypertension. However, several limitations should be acknowledged. First, measurement errors were inevitable due to self-reported socioeconomic information and hypertension based on a single blood pressure measurement(It needs to be noted that we conducted a separate analysis of participants who had already been diagnosed with hypertension, which demonstrated the stability of our results.). Second, despite accounting for important covariates in the analysis, the possibility of reverse causation and residual confounding (e.g., ambient air pollution [34]) cannot be fully eliminated. Third, the use of a BMI cutoff of 28 kg/m2 for obesity classification does not align with much of the international evidence, including the recommendations of the World Health Organization (30 kg/m2), which limits the extrapolation of results to some extent. Fourth, the cross-sectional design restricts the estimation of causal interpretations.

## Conclusion

Lower SES was positively associated with hypertension in our study. The highest risk of hypertension was observed in individuals with moderate SES and obesity, with stronger correlations between obesity and hypertension seen in this group. We found an independent and additive interaction effect of obesity and SES on the risk of hypertension. Thus, people with obesity, particularly those with moderate and low SES, should focus on reducing weight and waist circumference, and pay close attention to their blood pressure. Additionally, we recommend strengthening the adaptability of health education to the language and daily life of Tibetan residents, which can be achieved through the widespread dissemination of health knowledge, promotion of healthy lifestyles. Furthermore, the health administration departments should focus on deepening the management of chronic diseases and encouraging the signing of family doctor contracts to enhance their healthcare.

### Electronic supplementary material

Below is the link to the electronic supplementary material.

Supplementary Material 1

## Data Availability

Due to property rights protection, the data sets created and analyzed during the current study are not publically accessible, however they are available from the corresponding author upon justifiable request.

## Footnotes

1 Date Source:http://www.xizang.gov.cn/zwgk/zfsj/ndtjgb/202105/t20210520_202889.html. http://www.gov.cn/guoqing/2021-05/13/content_5606149.htm.
