# Supplementary material for: Associations of socioeconomic status and obesity with hypertension in tibetan adults in a Chinese plateau area
Source: BMC Public Health. 2023 Sep 21;23:1840. doi: 10.1186/s12889-023-15864-9 (PMC10515255; doi:10.1186/s12889-023-15864-9)
Supplement: Supplementary file 1 — Supplementary Material 1 [file 12889_2023_15864_MOESM1_ESM.docx]

Table S1 Indicators in models with different numbers of latent classes

| Number of class | AIC | BIC | VLMR-LRT | BLRT | Entropy | Sample proportion per class |
| --- | --- | --- | --- | --- | --- | --- |
| 1 | 62338.16 | 62393.78 | - | - | 1.00 | 7727 |
| 2 | 60867.73 | 60985.92 | <0.001 | <0.001 | 0.695 | 1231/6496 |
| 3 | 60539.78 | 60720.54 | <0.001 | <0.001 | 0.631 | 450/2236/5041 |
| 4 | - | - | - | - | 0.657 | 432/1860/1420/4015 |

Table S2 Associations of socioeconomic status and obesity with hypertension (non self-reported hypertension)

| Variables | Model 1 | Model 2 | Model 3 | Model 4 |
| --- | --- | --- | --- | --- |
| Socioeconomic status |  |  |  |  |
| High SES | 1.00(Reference) | 1.00(Reference) | 1.00(Reference) | 1.00(Reference) |
| Medium SES | 2.32(1.50, 3.60) | 2.26(1.46, 3.51) | 2.06(1.33, 3.19) | 2.11(1.26, 3.26) |
| Low SES | 1.80(1.21, 2.67) | 1.77(1.19, 2.62) | 1.62(1.09, 2.41) | 1.64(1.11, 2.44) |
| Obesity |  |  |  |  |
| No | 1.00(Reference) | 1.00(Reference) | 1.00(Reference) | 1.00(Reference) |
| Yes | 1.86(1.48, 2.33) | 2.66(2.01, 3.52) | 1.93(1.55, 2.39) | 1.82(1.37, 2.43) |

All models adjusted for age, sex, smoking status, alcohol drinking status, physical activity, sleep disorder, DASH score and hypertension family history. Model 1 was for general obesity by BMI ≥ 28; Model 2 was for general obesity by BMI ≥ 30; Model 3 was for abdominal obesity by waist circumference; Model 4 was for abdominal obesity by waist-to-height ratio.

Table S3 Associations of socioeconomic status and obesity with hypertension

| Variables | Model 1 | Model 2 | Model 3 | Model 4 |
| --- | --- | --- | --- | --- |
| Socioeconomic status |  |  |  |  |
| High SES | 1.00(Reference) | 1.00(Reference) | 1.00(Reference) | 1.00(Reference) |
| Medium SES | 2.12(1.54, 2.90) | 2.09(1.52, 2.87) | 1.89(1.38, 2.58) | 1.91(1.39, 2.61) |
| Low SES | 1.95(1.48, 2.57) | 1.94(1.47, 2.55) | 1.78(1.35, 2.34) | 1.78(1.35, 2.34) |
| Obesity |  |  |  |  |
| No | 1.00(Reference) | 1.00(Reference) | 1.00(Reference) | 1.00(Reference) |
| Yes | 1.82(1.54, 2.15) | 2.51(2.02, 3.12) | 1.79(1.53, 2.08) | 1.92(1.56, 2.36) |

All models adjusted for age, sex, smoking status, alcohol drinking status, physical activity, sleep disorder, DASH score, diabetes, and hypertension family history. Model 1 was for general obesity by BMI ≥ 28; Model 2 was for general obesity by BMI ≥ 30; Model 3 was for abdominal obesity by waist circumference; Model 4 was for abdominal obesity by waist-to-height ratio.
